# Concurrent Assessment of Sequential Auditory Event‐Related Potentials Using an Optimized Paired‐Stimulus Local–Global Paradigm

**DOI:** 10.1111/ejn.70423

**Published:** 2026-02-17

**Authors:** Chao Guo, Xiaoyu Wang, Zhaonan Ma, Xiao Yang, Fengyu Cong

**Affiliations:** ^1^ School of Biomedical Engineering, Faculty of Medicine Dalian University of Technology Dalian China; ^2^ Western Institute of Neuroscience Western University London Canada; ^3^ Department of Physiology and Pharmacology Western University London Canada; ^4^ Faculty of Information Technology University of Jyväskylä Jyväskylä Finland; ^5^ Key Laboratory of Social Computing and Cognitive Intelligence (Dalian University of Technology), Ministry of Education Liaoning China; ^6^ School of Software Engineering Dalian University Dalian China

**Keywords:** event‐related potentials (ERPs), local–global, mismatch negativity (MMN), P3b, voluntary attention

## Abstract

Comprehensive assessment of auditory processing is crucial for understanding perceptual and attentional functions, as well as detecting related deficits in clinical populations. Auditory event‐related potentials (ERPs) track key stages through time‐locked components that emerge from early sensory processing (P1–N1–P2 complex) and automatic deviance detection (mismatch negativity, MMN) to involuntary attention orienting (P3a) and voluntary attention engagement (P3b). However, current approaches predominantly focus on isolated ERP components demonstrated through group‐level statistical difference, whereas paradigms capable of capturing sequential components with high individual sensitivity remain scarce. Here, we optimized the local–global paradigm with paired‐stimulus design, strategically capturing pre‐attentive to voluntary processing by contrasting responses to within‐pair violations (local effect) versus across‐pair violations (global effect). We evaluated this paradigm in 30 healthy participants under active (target counting) and passive (visual distraction) conditions. Results demonstrated that both conditions reliably elicited complete pre‐attentive components (P1‐N1‐P2 and MMN) as confirmed by cluster‐based permutation tests, achieving 30/30 individual‐level sensitivity validated through intrasubject classification analysis. Furthermore, comparison between active and passive conditions revealed significant differences specifically in the 272–392 ms and 272–400 ms window (*p* < 0.05) under two levels of global deviants. This contrast successfully dissociated voluntary from involuntary attention with 86.67% and 93.33% individual sensitivity, respectively. Moreover, the active‐passive discrimination depended primarily on the number of epochs sampled (*p* < 0.001) rather than the number of sensors used (*p* > 0.05). These findings validate our paired‐stimulus local–global paradigm as a reliable approach for assessing sequential auditory ERPs, offering significant advantages with potential applications in clinical evaluation of perceptual and attentional impairments.

## Graphical abstract

A novel paired‐stimulus local–global paradigm was validated for concurrent assessment of auditory processing from early sensory processing (P1–N1–P2) and automatic deviance detection (MMN) to involuntary attention (P3a) and voluntary attention (P3b). Task manipulation effectively dissociates involuntary from voluntary attention. Robust individual‐level sensitivity underscores the paradigm's utility for efficient single‐session evaluations.

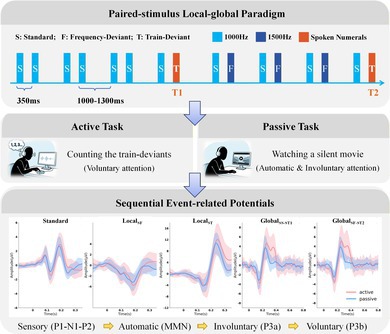

**Abbreviations**
DOC: disorders of consciousness
EEG: electroencephalography
ERP: event‐related potential
ICA: independent component analysis
ML: machine learning
MMN: mismatch negativity
RS: resting state
SNR: signal‐to‐noise ratio
SOA: stimulus onset asynchrony
SVM: support vector machine

## 1 Introduction

Auditory processing unfolds through sequential stages, from early sensory encoding and automatic deviance detection to involuntary attention orienting and voluntary attention engagement, providing a framework for evaluating perceptual and attentional functions (Nelken et al. 2014; Peterson et al. 2025; Price and Moncrieff 2021). The integrity of this hierarchical processing is crucial, as disruptions at any stage can degrade diagnostic sensitivity through cascading propagation of neural deficits. Specifically, compromised sensory gating precludes reliable deviance detection, whereas impaired automatic processing obscures conscious attention allocation (Comanducci et al. 2020; Sergent et al. 2021). Consequently, precise localization of impairment along the auditory processing pathway becomes essential for reliable neurocognitive assessment. This need is particularly important in disorders of consciousness (DOC), where behavioral responses are absent or ambiguous, yet preserved auditory processing may indicate covert consciousness undetectable through clinical examination (Sitt et al. 2014). Objective neurophysiological measurements thus provide a valuable window into these patients' cognitive capacities.

Auditory event‐related potentials (ERPs) offer millisecond‐precision tracking of sequential neural processes through distinct time‐locked components. Early components reflect automatic and pre‐attentive processing that occurs without conscious awareness. The P1–N1–P2 complex (50–200 ms) emerges as an obligatory response to any auditory stimulus, representing basic sensory registration (Näätänen and Picton 1987). In contrast, mismatch negativity (MMN, 100–250 ms) is specifically elicited when infrequent deviant sounds violate the regularity established by repetitive standards, indexing automatic change detection (Garrido et al. 2009; Näätänen et al. 2007). These components can be reliably recorded during passive listening, when participants' attention is directed elsewhere through reading or visual tasks, and persist even in unresponsive patients with preserved sensory function. This independence from conscious attention confirms their automatic nature and establishes their value as objective markers of intact early auditory processing (Fischer et al. 2010; Näätänen et al. 2012).

Following these pre‐attentive components, later ERPs reveal how attention modulates auditory processing. The P3a component (250–350 ms) reflects involuntary attention orienting, emerging when salient or unexpected stimuli automatically capture attention regardless of task demands (Atienza et al. 2001). This frontocentral response occurs even during passive conditions, distinguishing it from the P3b component (300–500 ms), which manifests exclusively during active target detection tasks (Rutiku et al. 2024). Unlike P3a's automatic emergence, P3b requires voluntary attention allocation and reflects conscious stimulus evaluation, appearing over centroparietal regions (Barry et al. 2016; Polich 2007). This functional dissociation between P3a and P3b, demonstrable by comparing active counting tasks with passive listening conditions, provides crucial insights into attentional hierarchies. In clinical populations, particularly in patients with DOC, preserved P3a with absent P3b indicates intact bottom‐up attention orienting despite impaired conscious integration functions (Kotchoubey et al. 2005; Sergent et al. 2005). Therefore, paradigms capable of reliably eliciting and dissociating these attention‐related components at the individual level are essential for accurate assessment of residual cognitive function.

These sequential components, from pre‐attentive to voluntary processing, collectively offer a comprehensive view of auditory cognitive function. However, current paradigms face significant limitations in harnessing this diagnostic potential for clinical assessment due to a fragmented assessment approach. Typically, different components are evaluated in separate experimental sessions tailored to specific arousal thresholds (Chennu and Bekinschtein 2012). For instance, the passive oddball with simple tones is applied for pre‐attentive responses (P1–N1–P2 and MMN), whereas novelty paradigms using semantically rich stimuli (like the subject's own name or spoken words) are used for P3a, and active target tasks for P3b, thereby facilitating the engagement of residual automatic and attentional resources (Annen et al. 2020; Barry et al. 2016; Chennu et al. 2013; Chennu and Bekinschtein 2012; Garrido et al. 2009; Merchie and Gomot 2023; Morlet et al. 2017; Sculthorpe‐Petley et al. 2015; Wang et al. 2024; Wu et al. 2020). This fragmentation proves particularly problematic in DOC patients, whose fluctuating arousal states make multiple testing sessions unreliable or impossible (Edlow et al. 2021).

To address efficiency, specialized paradigms such as the roving standard paradigm have been developed and further refined to capture the full hierarchy of auditory responses. This paradigm controls for physical stimulus differences by continuously changing the standard stimulus. This design elegantly isolates repetition positivity from genuine prediction error, reflecting sensory adaptation and memory trace formation (Garrido et al. 2008; McCleery et al. 2019; Wang et al. 2025). However, although excellent for probing low‐level sensory learning, the roving paradigm typically lacks a stable global rule required to elicit the P3b component, rendering it insufficient for assessing higher‐order voluntary attention. Similarly, multi‐feature paradigms have been developed to record profiles of multiple MMNs in a short time by alternating various deviant features (Li et al. 2025; Näätänen et al. 2012; Pakarinen et al. 2009). Although highly effective for mapping pre‐attentive sensory discrimination, these paradigms typically focus on the automatic level of processing and do not explicitly engage or assess the hierarchical transition to voluntary executive control (P3b).

Conversely, selective attention and classic oddball paradigms successfully isolate voluntary attention mechanisms. Recent studies utilizing spatial or sequential oddball tasks have demonstrated robust P3b responses associated with conscious change detection and cognitive control in both real and virtual environments (Fogarty et al. 2019; Lewald et al. 2018; Stodt et al. 2025). However, these paradigms often confound local physical deviance with global task relevance (i.e., the deviant stimulus is inherently the target), making it challenging to disentangle bottom‐up saliency from top‐down rule processing within a single sequence. Furthermore, they typically assess voluntary attention in isolation from pre‐attentive processing, failing to capture the dynamic interaction between these levels.

The local–global paradigm partly addresses this fragmentation and confounding by embedding multiple regularity levels within a single auditory sequence. Local regularities (within‐trial acoustic patterns) probe pre‐attentive processing, whereas global regularities (across‐trial sequence violations) engage higher‐order conscious detection (Bekinschtein et al. 2009; King et al. 2013). Crucially, unlike multi‐feature or standard oddball designs, this paradigm allows for the concurrent assessment of the full processing hierarchy from early sensory components to automatic change detection (MMN) and voluntary rule processing (P3b) in one continuous session. By orthogonally manipulating local physical deviance and global task relevance, it provides a precise dissociation between automatic and attention‐dependent mechanisms. Despite these theoretical advantages, the clinical utility of the local–global paradigm has been constrained by challenges in individual‐level sensitivity. Although robust at the group level, few studies have systematically validated its diagnostic accuracy in single subjects compared to optimized oddball tasks (Rutiku et al. 2024), which is essential for reliable clinical diagnosis.

To address these limitations, we developed an optimized paired‐stimulus local–global paradigm designed to temporally separate local (within‐pair deviations) and global (across‐pair violations) effects under both active and passive conditions. By leveraging the fundamental mechanism of immediate trace comparison, this design significantly increases trial density and efficiency, enabling the robust extraction of the sequential ERP within a short timeframe. Our primary goal was to validate that this high‐efficiency design could reliably elicit the full sequence of auditory ERPs with high individual sensitivity in a single session and determine whether task‐based contrasts can effectively separate P3a from P3b in individual recordings. Specifically, we hypothesized that (1) robust early sensory (P1–N1–P2 complex), pre‐attentive (MMN), and involuntary attention (P3a) components would be elicited across individuals regardless of tasks; (2) the active–passive contrast would selectively modulate the P3b, thereby dissociating voluntary from involuntary attentional processes; and (3) the reliable detection of the voluntary attention effect would be more critically dependent on temporal sampling (the number of trials) than spatial resolution (the number of sensors). Demonstrating this research provides essential groundwork for translating ERP‐based consciousness assessment into clinical practice, particularly for intensive care patients with fluctuating arousal states.

## 2 Materials and Methods

### 2.1 Participants

Thirty adult undergraduate participants (mean age 24.70 ± 1.37; 19 male) were recruited from Dalian University of Technology. All participants reported normal hearing, and none had a history of neurological or psychiatric illnesses. The study was approved by the Ethics Committee of Dalian University of Technology (DUTFM250630‐01). All participants gave written informed consent after obtaining knowledge of this experiment.

### 2.2 Stimulation Paradigm

**FIGURE 1:**
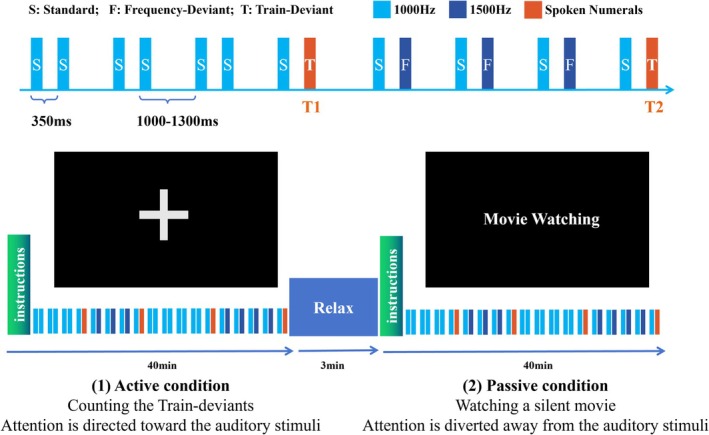
Experimental design of the paired‐stimulus local–global paradigm. Upper panels: Schematic illustration of the stimulation sequence. The paradigm consists of paired stimuli where the first sound in each pair is fixed as 1000 Hz standard tone (S, blue), followed by either another 1000 Hz standard tone (S, blue), 1500 Hz frequency‐deviant tone (F, navy blue), or train‐deviant stimulus (T, red). The stimulus onset asynchrony between sounds within a pair was fixed at 350 ms, whereas pairs were separated by a jittered interval ranging from 1000 to 1300 ms. Local deviants were defined by acoustic changes within pairs (S–F and S–T), whereas global deviants were defined by pattern violations across sequences of pairs. Bottom panels: Illustration of the two attentional conditions. In the active attention condition, participants were instructed to mentally count the train‐deviant target stimuli (S–T pairs) while ignoring other stimuli. In the passive attention condition, participants watched a silent movie (“Modern Times”), whereas the identical auditory sequence was presented, allowing for comparison between voluntary and automatic processing of auditory regularities.

The study employed a paired‐stimulus local–global paradigm as illustrated in Figure 1. Three types of stimuli were used in the experiment, categorized into pure tones and spoken numerals. The first category consisted of pure tones, serving as the standard (S; 1000 Hz) and frequency‐deviant (F; 1500 Hz) stimuli. Both tone types were generated with a duration of 200 ms, including 20 ms rise and fall times. The second category, the train‐deviant stimuli (T), comprised spoken numerals ranging from “one” to “nine.” These stimuli were synthesized using the Microsoft Simplified Chinese text‐to‐speech engine via Adobe Audition. Acoustically, they featured a synthesized female voice with natural intonation and a fundamental frequency centered approximately at 220 Hz. To ensure consistent loudness and perceptibility, the audio files were processed using a speech volume leveler with a target volume level of −10 dB and a leveling amount of 100%. All speech stimuli were edited to match the 200 ms duration of the pure tones.

In terms of the paired‐stimulus, the first stimulus in each pair was fixed as S, resulting in three combinations with the following probabilities: S–S pairs (41.11%), S–F pairs (40.00%), and S–T pairs (18.89%). Within this paradigm, local deviants were defined as any pair where the second stimulus differed from the first one, including Local_SF_ (S–F pairs) and Local_ST_ (S–T pairs). Global deviants were defined as violations of established patterns across sequences of pairs. To distinguish between specific sequence contexts, we categorized the global deviations into two subtypes: (1) T1, defined as the trailing stimulus of an S–T pair that disrupted a global regularity established by repeated S–S pairs; and (2) T2, defined as the trailing stimulus of an S–T pair that disrupted a global regularity established by repeated S–F pairs. These S–T pairs were interspersed pseudo‐randomly throughout the sequence, with the constraint that any two S–T pairs were separated by at least three intervening pairs (either S–S or S–F).

The stimulus onset asynchrony (SOA) between sounds within a stimulus pair was fixed at 350 ms. Between pairs, a jittered interval ranging from 1000 to 1300 ms (with 50 ms steps) was implemented to enhance ERP signal quality by preventing anticipatory responses (Luck 2014). The experiment included both active and passive sessions, each session comprising a total of 1380 stimuli pairs, with the first 30 S–S pairs serving to establish regularity (excluded from analysis). Of the remaining 1350 pairs analyzed, there were 555 S–S pairs, 540 S–F pairs (all functioning as Local_SF_), and 255 S–T pairs (all serving as Local_ST_). For the global effect analysis, these included 121 instances of Global_SS‐ST1_ (S–T pairs disrupting S–S regularity) and 134 instances of Global_SF‐ST2_ (S–T pairs disrupting S–F regularity).

The experiment consisted of two sessions designed to contrast voluntary and involuntary attention processing. During both sessions, participants sat in a comfortable chair in a sound‐attenuated booth and received instructions via text displayed on a computer screen. The auditory stimulation remained identical across both conditions, with stimuli delivered through dual‐channel earphones. In the active attention condition, participants were instructed to perform a target detection task by mentally counting the occurrence of train‐deviant stimuli (spoken numerals ranging from “one” to “nine”) regardless of the preceding sequence context and reporting their total at the end of the session. In the passive attention condition, participants were instructed to focus on watching a silent movie (“Modern Times”) displayed on a laptop while ignoring the auditory stimuli (Figure 1, bottom panels). Each session lasted approximately 40 min and was implemented using the Psychophysics Toolbox Version 3 (Brainard 1997).

### 2.3 EEG Recordings and Preprocessing

EEG data were recorded with a 64‐channel ANT recording device (ANT Neuro) configured by eego amplifier and Ag/AgCl electrodes according to the 10/20 International System. The sampling rate was 1000 Hz, and the electrode impedance of all subjects was kept below 20 kΩ. Data were referenced online at the CPz electrode.

The recorded EEG data were preprocessed using the EEGLAB toolbox (Delorme and Makeig 2004). Firstly, visual inspection was conducted to remove significant artifacts caused by body movements, amplifier clipping, or bursts of EEG activity. Channels with excessive noise or poor signal quality were identified and replaced using spherical spline interpolation. No more than 5% of channels were interpolated for any participant. Then, the inspected data underwent sequential filtering with a 50‐Hz notch filter, 1‐Hz high‐pass filter, and 30‐Hz low‐pass filter in order. The filtered data were re‐referenced offline to the mean potential of two mastoid sites (M1/M2) and down‐sampled to 250 Hz. Artifacts were corrected using independent component analysis (ICA) (Lee et al. 1999). We utilized the ICLabel plugin (Pion‐Tonachini et al. 2019) to automatically classify independent components. Components identified as eye movements, muscle activity, and channel noise with a probability estimate exceeding 90% were removed. Finally, the Fz and Pz electrodes were selected for the subsequent ERP analysis.

### 2.4 Data Analysis

The analysis followed a stepwise approach to evaluate both group‐level ERP characteristics and individual‐level sensitivity. We first examined whether this paradigm reliably elicited the target ERP components at the group level. We then quantified the individual‐level sensitivity of each component using machine learning (ML) techniques. Finally, we compared active versus passive conditions to differentiate involuntary from voluntary attentive processing at both group and individual levels.

#### 2.4.1 Group‐Level Characteristics Analysis

We analyzed three aspects of brain responses to evaluate auditory ERP components. First, the obligatory P1–N1–P2 complex was examined by comparing the response elicited by the first standard stimulus (S) in each pair against the response derived from the pre‐stimulus resting state (RS). For S, we extracted a 450‐ms epoch including a 100‐ms pre‐stimulus period, with baseline correction using the −100–0 ms window. For RS, we used the 450‐ms interval immediately preceding S onset (−450–0 ms), with baseline correction using the −450 to −350 ms window. Standard S and RS trials with voltages exceeding ±100 μV were concurrently rejected for the guarantee that the number of them was matched, resulting in 1331 ± 24 accepted trials per participant for S and RS, respectively.

Subsequently, local effects were examined to identify MMN/P3a components by comparing the responses to deviant stimuli and standard stimuli within individual stimulus pairs. We analyzed two types of local effects: (1) Local_SF_ effect, derived from S–F pairs by comparing the frequency deviant (F) to the preceding standard tone (S), and (2) Local_ST_ effect, derived from S–T pairs by comparing the train‐deviant (T) to the preceding standard tone (S). We used the same epoch extraction and artifact rejection criteria as in the P1–N1–P2 analysis. After artifact rejection, we retained epochs including 530 ± 11 S–F trials per participant for Local_SF_ analysis and 250 ± 6 S‐T trials per participant for Local_ST_ analysis.

Third, global effects were analyzed to disentangle P3a and P3b components by examining responses to global pattern violations across stimulus pairs. We assessed two types of global effects: (1) Global_SS‐ST1_ effect, derived from the S–S context, comparing the response to the train‐deviant (T) in S–T pairs against the response to the second standard tone (S) in the preceding S–S pair, and (2) Global_SF‐ST2_ effect, derived from the S–F context, comparing the response to the train‐deviant (T) in S–T pairs against the response to the frequency‐deviant tone (F) in the preceding S–F pair. For global analyses, we extracted 900‐ms epochs time‐locked to the onset of the second stimulus in each pair, including a 100‐ms pre‐stimulus baseline period. Using the same artifact rejection criteria, we retained epochs with 118 ± 4 trials per participant for Global_SS‐ST1_ analysis and 130 ± 5 trials per participant for Global_SF‐ST2_ analysis.

At the group level, ERP components were characterized by a cluster‐based permutation approach with threshold‐free cluster enhancement. This analysis was performed on the individual‐averaged waveforms, with paired *t*‐tests conducted across participants. The empirical distribution was established through 1000 random permutations, with the alpha level set at 0.05 (two‐tailed). Significant clusters were identified when at least two neighboring electrodes showed significant effects at the same time point, with an additional requirement that effects persist for at least 20 ms to correct for multiple comparisons in the time domain. In Section 3, we report the time range (onset/offset) of significant clusters and their corresponding statistical values at the representative electrodes (global effects were reported at the Pz electrode, whereas other ERPs were reported at the Fz electrode).

#### 2.4.2 Individual‐Level Sensitivity Analysis

We implemented a ML approach to assess the individual‐level sensitivity of each ERP component. The individual condition comparison and epoch extraction method for three aspects of brain responses were similar to the group‐level characteristics analysis. For P1–N1–P2 complex, two conditions were the first standard stimulus (S) in each pair and RS preceding S onset. For local effects, two conditions were standard and deviant stimuli within pairs. For global effects, two conditions were the second stimulus in train‐deviant pairs (T) and the second stimulus in pairs preceding S–T. Moreover, another comparison was between active and passive conditions under global effects. We focused on 0–300 ms post‐stimulus period for P1–N1–P2 complex and local effects and 0–400 ms post‐stimulus period for global effects.

To enhance signal‐to‐noise ratio (SNR) while maintaining sufficient samples for classification, we reorganized the epoch data (trials × channels × time points). Each participant's trials were divided into 50 non‐overlapping blocks, with consecutive trials averaged within each block. For instance, 200 trials were grouped into 50 blocks of 4 trials each, yielding 50 averaged samples per condition. This resulted in balanced datasets of 100 samples (50 per condition) for binary classification.

The classification pipeline employed support vector machine (SVM) approaches with default hyperparameters and fivefold cross‐validation to quantify component‐specific discriminability of ERPs. Two distinct binary classification tasks were systematically conducted: (1) S and RS for P1–N1–P2 complex, (2) standards and deviants for local and global effects, and (3) active and passive conditions for condition comparison. Model performance was quantified using classification accuracy, computed as the mean proportion of correctly classified trials across all cross‐validation folds. To establish significance, we implemented a permutation framework with 1000 iterations using true labels to generate the performance distribution and 1000 iterations with randomly shuffled labels to create the null distribution. Components were considered reliably detected when classification accuracy exceeded the 95th percentile of the null distribution.

#### 2.4.3 Active–Passive Classification Across Varying EEG Configurations

To establish whether voluntary attention detection could be achieved with reduced recording demands, we evaluated how classification performance changed when reducing either spatial coverage or temporal sampling. We tested 16 configurations in a 4 × 4 factorial design: sensor montages of 8, 16, 32, and 64 channels (selected to preserve 10–20 system topology) crossed with 25%, 50%, 75%, and 100% of available epochs.

Each configuration underwent SVM classification using the pipeline described in Section 2.4.2, with active versus passive conditions serving as classification targets during global effect processing. Notably, because 25% of the available epochs are insufficient to provide 50 samples per condition, we used the original number of epochs as samples. To ensure robust performance estimates, we repeated fivefold cross‐validation 500 times per configuration. The relationship between classification accuracy and recording parameters was assessed using Spearman's rank correlation, separately examining the effects of electrode count and epoch percentage. Statistical significance was determined through percentile bootstrap with 1000 iterations (Efron and Tibshirani 1994), with 95% confidence intervals computed from the bootstrap distribution.

## 3 Results

### 3.1 Validation of Component Elicitation Across Conditions

#### 3.1.1 P1–N1–P2 Complex

**FIGURE 2:**
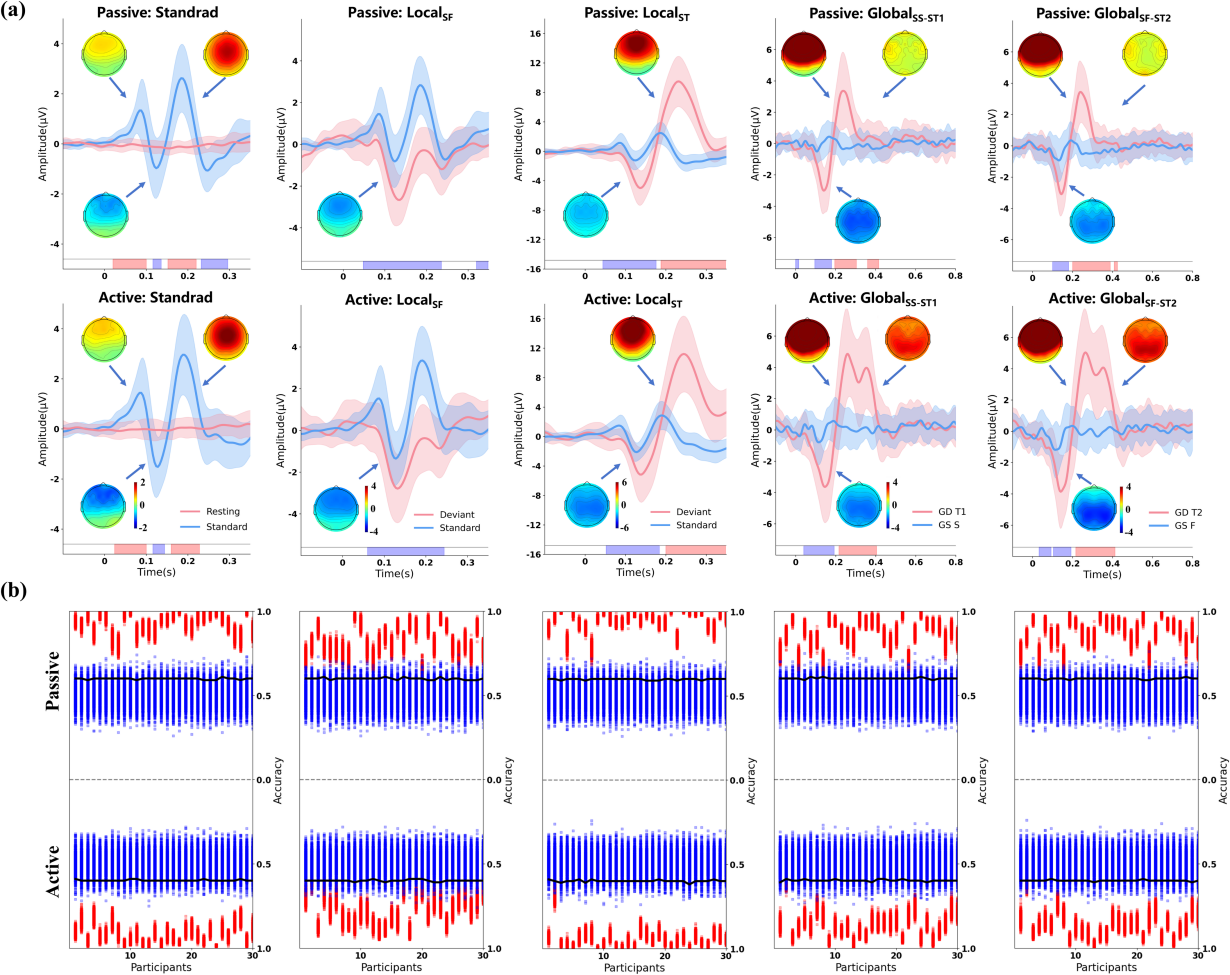
ERP analysis of the local and global effect. (a) Group‐level waveforms on deviant (red line) and standard (blue line) conditions across the local and global effect in passive and active condition. For P1–N1–P2 complex, the contrast conditions are standard (blue line) and resting state (red line). Shaded areas represent standard error across participants. The color rectangles below the waveform represent the significant difference between conditions (*p* < 0.05, cluster‐corrected), with color intensity indicating the polarity of difference waves. Scalp topographies display the mean voltage amplitude of the difference waves averaged across the significant time window indicated by the color rectangles areas in the passive and active conditions, with a specific exception for the global effect. To distinctively characterize the P3 subcomponents, topographies were computed over fixed time windows of 200–300 ms for the P3a and 300–400 ms for the P3b. Individual color scales are used for each effect to indicate the polarity and amplitude of the difference waves. “GD T1” and “GS S” respectively represent global deviants (T1, train stimuli) and global standard (S, 1000 Hz). “GD T2” and “GS F,” respectively, represent global deviants (T2, train stimuli) and global standard (F, 1500 Hz). (b) Individual‐sensitivity detection for five effects in passive and active conditions. Red regions represent the accuracy distribution of true label. Blue regions represent the accuracy distribution of randomly permuted label. Black lines represent individual significance thresholds (*p* < 0.001, Bonferroni‐corrected).

For the P1–N1–P2 complex, group‐level comparison of standard stimuli with resting state at the Fz electrode revealed reliable component elicitation in both passive and active conditions (Figure 2a, left panel). Three distinct clusters emerged with consistent temporal profiles: a positive P1 cluster (passive: 20–100 ms, *p* < 0.05, peak *t* = 124 at 76 ms; active: 24–100 ms, *p* < 0.05, peak *t* = 116 at 76 ms), followed by a negative N1 cluster (passive: 116–136 ms, *p* < 0.05, peak *t* = 34 at 124 ms; active: 116–144 ms, *p* < 0.05, peak *t* = 75 at 128 ms), and a positive P2 cluster (passive: 152–220 ms, *p* < 0.05, peak *t* = 300 at 184 ms; active: 160–228 ms, *p* < 0.05, peak *t* = 277 at 188 ms).

At the individual level, the SVM classification between standard stimuli and resting state demonstrated exceptional detection sensitivity (Figure 2b, left panel). All 30 participants achieved significant discrimination in both passive (mean accuracy = 0.92 ± 0.05) and active (mean accuracy = 0.93 ± 0.05) conditions, yielding a 100% individual detection rate (*p* < 0.001). These results confirm that our paradigm reliably captured early sensory processing in every tested participant.

#### 3.1.2 Local Effects

Group‐level analysis of within‐pair variations revealed robust MMN and P3a components at Fz electrode across both passive and active conditions (Figure 2a, second and third columns). For the Local_SF_ effect (frequency‐deviants), cluster‐based permutation tests revealed negative MMN clusters in both passive (48–236 ms, *p* < 0.05, peak *t* = 347 at 148 ms) and active (60–244 ms, *p* < 0.05, peak *t* = 369 at 196 ms) conditions, confirming reliable detection of acoustic feature changes. For the Local_ST_ effect (train‐deviants), the paradigm elicited a more complex response pattern comprising both MMN (passive: 44–176 ms, *p* < 0.05, peak *t* = 358 at 144 ms; active: 52–184 ms, *p* < 0.05, peak *t* = 160 at 140 ms) and subsequent P3a components (passive: 200–348 ms, *p* < 0.05, peak *t* = 564 at 232 ms; active: 192–348 ms, *p* < 0.05, peak *t* = 355 at 248 ms). This MMN‐P3a complex indicates that train deviants engaged both automatic deviance detection and involuntary attention orienting mechanisms.

At the individual level, the SVM classification between within‐pair deviant and standard stimuli demonstrated high detection sensitivity (Figure 2b, second and third columns). For Local_SF_, 29/30 participants manifested significant MMN responses (*p* < 0.001), with one participant (participant #2, mean accuracy = 0.57 ± 0.04) in the passive condition falling below the significance threshold (0.60). In contrast, all participants exhibited significant MMN responses in the active condition (*p* < 0.001). For Local_ST_, all participants demonstrated significant MMN‐P3a complex responses in both passive (mean accuracy = 0.94 ± 0.06) and active (mean accuracy = 0.95 ± 0.05) conditions (*p* < 0.001), yielding a 100% individual detection rate. These results confirm that within‐pair variations reliably elicited automatic change detection (MMN) and involuntary attention orienting (P3a) components across participants.

#### 3.1.3 Global Effects

For the Global_SS‐ST1_ effect (train‐deviants disrupting S–S regularity), cluster‐based permutation tests at Pz electrode revealed an early negative cluster in both passive and active conditions (passive: 96–180 ms, *p* < 0.05, peak *t* = 266 at 148 ms; active: 40–192 ms, *p* < 0.05, peak *t* = 219 at 152 ms), followed by a positive cluster that differed between conditions (passive: 196–304 ms, *p* < 0.05, peak *t* = 268 at 248 ms; active: 216–404 ms, *p* < 0.05, peak *t* = 309 at 284 ms). For the Global_SF‐ST2_ effect (train‐deviants disrupting S‐F regularity), a similar pattern emerged with an early negative cluster (passive: 100–180 ms, *p* < 0.05, peak *t* = 224 at 148 ms; active: 104–192 ms, *p* < 0.05, peak *t* = 215 at 164 ms) and a subsequent positive cluster (passive: 200–388 ms, *p* < 0.05, peak *t* = 377 at 248 ms; active: 216–412 ms, *p* < 0.05, peak *t* = 309 at 280 ms). Notably, the positive clusters extended significantly longer in the active condition.

At the individual level (Figure 2b, fourth and fifth columns), for Global_SS‐ST1_, 29/30 participants showed significant discrimination in the passive condition (mean accuracy = 0.87 ± 0.06, *p* < 0.001), with participant #2 (mean accuracy = 0.58 ± 0.03) falling below threshold (0.6). All participants achieved significant discrimination in the active condition (mean accuracy = 0.87 ± 0.07, *p* < 0.001), yielding a 100% detection rate. For Global_SF‐ST2_, all participants demonstrated significant responses in both passive (mean accuracy = 0.84 ± 0.07) and active (mean accuracy = 0.88 ± 0.08) conditions (*p* < 0.001). The high individual detection rates confirm the robustness of global effects elicitation across participants.

### 3.2 Differential Task Effects Across ERP Components

**FIGURE 3:**
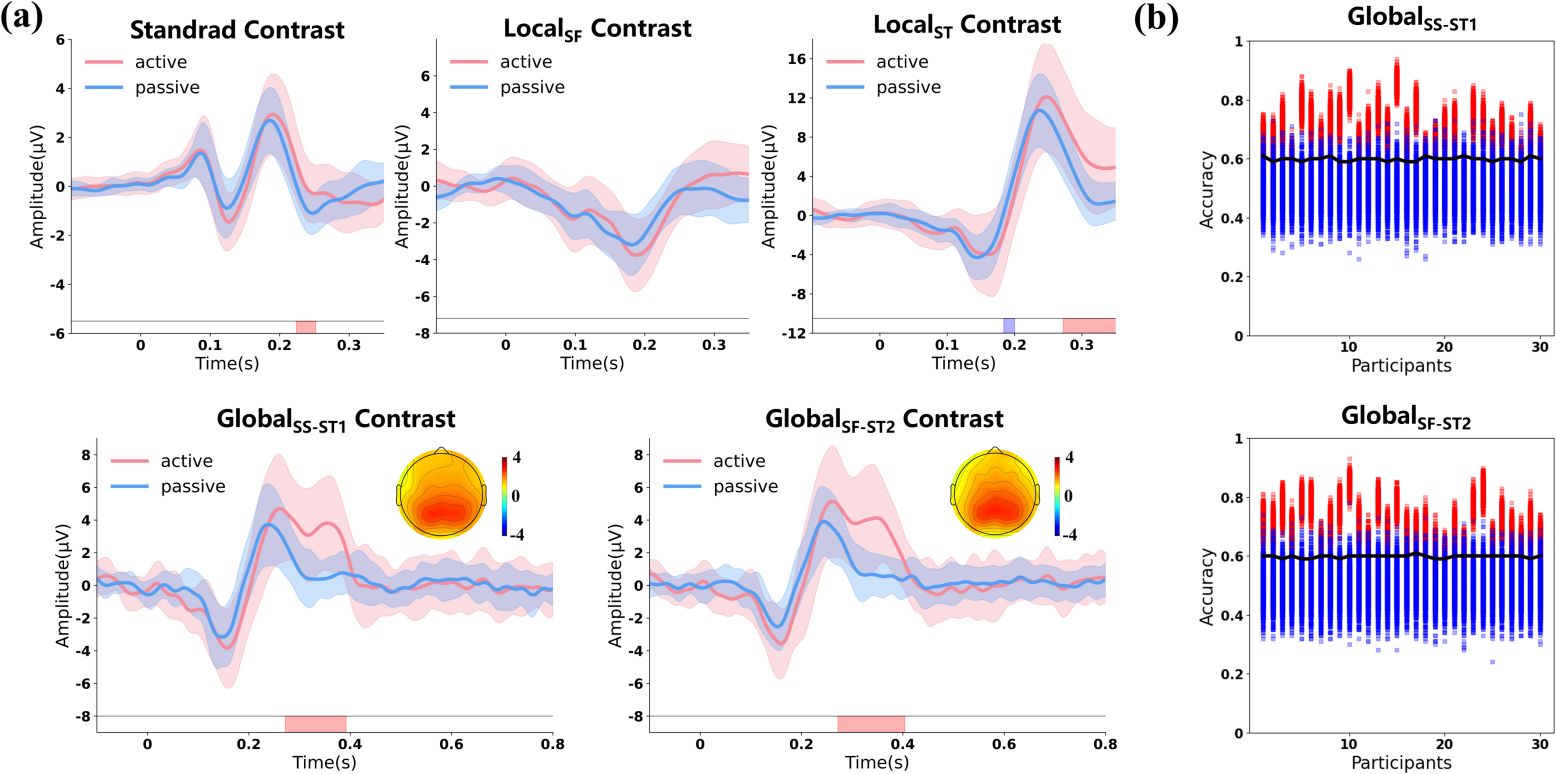
Contrast on ERP characteristics between passive and active conditions. (a) Group‐level difference waveforms between active (red line) and passive (blue line) conditions across the local and global effect. Shaded areas represent standard error across participants. The color rectangles below the waveform represent the significant difference between different conditions (*p* < 0.05, cluster‐corrected). Scalp topographies display the mean voltage amplitude of the difference waves averaged across the significant time window indicated by the color rectangles areas in the passive and active conditions under two levels of global deviants. (b) Individual sensitivity of the contrast between passive and active conditions under two levels of global deviants. Red regions represent the accuracy distribution of true label. Blue regions represent the accuracy distribution of randomly permuted label. Black lines represent individual significance thresholds (*p* < 0.001, Bonferroni‐corrected).

To dissociate voluntary from involuntary attention processing, we directly compared ERP responses between active and passive conditions across all experimental contrasts. Cluster‐based permutation tests demonstrated differential task effects across ERP components (Figure 3a). For early sensory and automatic processing, no significant differences emerged between active and passive conditions for the P1–N1–P2 complex or for MMN elicited by frequency deviants (Local_SF_), confirming their pre‐attentive nature. However, for Local_ST_ (numerical deviants), significant differences appeared in both the MMN window (184–200 ms, *p* < 0.05, peak *t* = 32 at 192 ms) and the P3a window (272–352 ms, *p* < 0.05, peak *t* = 53 at 344 ms). Most critically, for global effects, robust differences emerged specifically in the P3b time window for both Global_SS‐ST1_ (272–392 ms, *p* < 0.05, peak *t* = 85 at 348 ms) and Global_SF‐ST2_ (272–400 ms, *p* < 0.05, peak *t* = 93 at 364 ms). These extended positive clusters in the active condition reflect the engagement of voluntary attention processing (P3b) beyond involuntary orienting (P3a).

At the individual level (Figure 3b), classification between active and passive conditions yielded high sensitivity specifically for global contrasts. For Global_SS‐ST1_, 26/30 participants demonstrated significant between‐condition discriminability (mean accuracy = 0.70 ± 0.06, *p* < 0.001), achieving 86.67% detection sensitivity. For Global_SF‐ST2_, 28/30 participants showed significant discrimination (mean accuracy = 0.71 ± 0.06, *p* < 0.001), yielding 93.33% detection sensitivity. These results confirm that whereas early automatic components remained stable across task demands, later attentional components showed significant task‐related modulation, particularly in the P3 time window.

### 3.3 Optimal Recording Parameters for Voluntary Attention Detection

**FIGURE 4:**
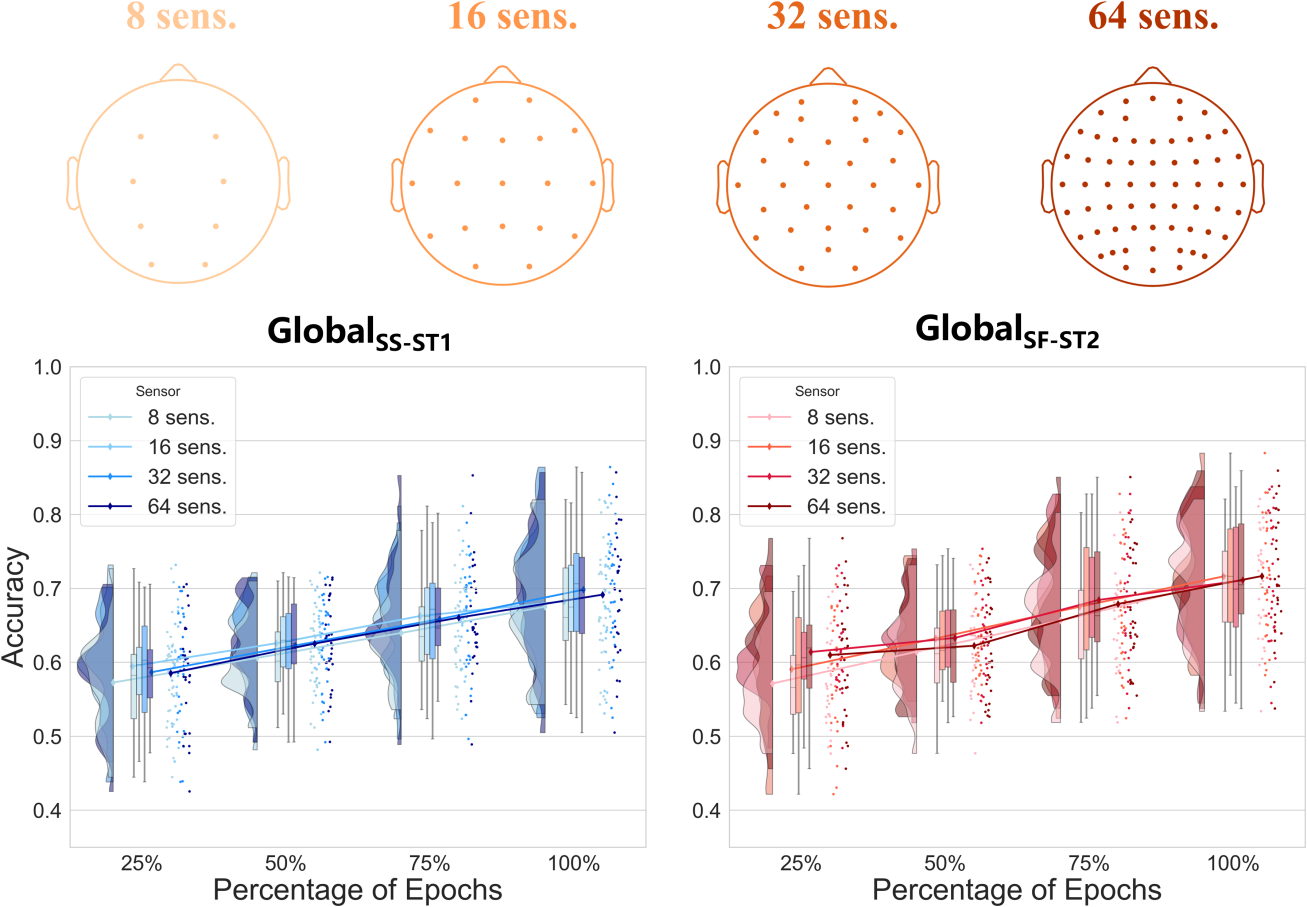
Performance of active–passive discrimination across different EEG configurations under two levels of global deviants Upper panels: Electrode montages of 8, 16, 32, and 64 sensors. Sensors were selected such that they approximated realistic EEG caps respecting the international 10–20 system. Bottom panels: Blue lines represent Global_SS‐ST1_ effects. Red lines represent Global_SF‐ST2_ effects. Data are presented as mean with error bars indicating standard error. There are 16 configurations in a 4 × 4 factorial design: 8, 16, 32, and 64 sensors crossed with 25%, 50%, 75%, and 100% of available epochs (S = 1000 Hz standards; F = 1500 Hz frequency‐deviants; T = train‐deviants; sens. = sensors).

To establish practical recording guidelines, we systematically evaluated how sensor density and epoch number affect the detection of voluntary attention engagement through active–passive discrimination. Analysis across 16 different EEG configurations revealed that discrimination performance was primarily driven by temporal sampling rather than spatial coverage (Figure 4). For Global_SS‐ST1_, classification accuracy showed strong positive correlation with epoch percentage (rho_Spearman_ = 0.97, 95% CI: 0.92–0.98, *p* < 0.001) but no significant correlation with sensor count (rho_Spearman_ = 0.11, 95% CI: −0.42–0.59, *p* > 0.05). Similarly, for Global_SF‐ST2_, performance correlated significantly with epoch percentage (rho_Spearman_ = 0.97, 95% CI: 0.91–0.98, *p* < 0.001) but not with sensor count (rho_Spearman_ = 0.17, 95% CI: −0.39–0.63, *p* > 0.05). Interestingly, the 32‐sensor (mean accuracy = 0.65 ± 0.04) configuration achieved comparable performance to the full 64‐sensor (mean accuracy = 0.65 ± 0.04) setup across both global contrasts. These results demonstrate that sufficient temporal sampling is more critical than extensive spatial coverage for reliably detecting voluntary attention signatures.

## 4 Discussion

This study introduced and validated a paired‐stimulus local–global paradigm that addresses the fundamental challenge of comprehensively assessing sequential auditory processing stages while ensuring high individual detection sensitivity within a single experimental session. Our paradigm successfully elicited target components from early sensory processing (P1–N1–P2 complex), automatic deviance detection (MMN), and involuntary attention orienting (P3a) to voluntary attention engagement (P3b), achieving exceptional individual‐level detection sensitivity (details in Table 1). Most critically, by contrasting active and passive conditions, we achieved 86.67% and 93.33% individual‐level sensitivity in dissociating voluntary from involuntary attention processing for two types of global deviants. Furthermore, we demonstrated that temporal sampling (number of epochs) rather than spatial coverage (number of sensors) primarily determines the reliability of voluntary attention detection, suggesting practical recording parameters for future implementation.

**TABLE 1:** Individual sensitivity of ERP elicitation across conditions.

Conditions	Standard (P1–N1–P2)	Local_SF_	Local_ST_	Global_SS‐ST1_	Global_SF‐ST2_
Passive	30/30	29/30	30/30	29/30	30/30
Active	30/30	30/30	30/30	30/30	30/30
Passive vs. active	—	—	—	26/30	28/30

Our paradigm's ability to reliably elicit the P1–N1–P2 complex with 100% detection sensitivity across all participants represents a significant methodological achievement. The temporal and spatial characteristics of these components align well with established literature (Näätänen and Picton 1987; Picton et al. 1974). We attribute this high sensitivity to two key design features. First, the fixed first‐standard design within all stimulus pairs ensured sufficient trial numbers (1331 ± 24 per participant) to enhance SNR for robust classification. Second, using the pre‐stimulus period as baseline (−450–0 ms before first stimulus) provided clear separation between stimulus‐driven responses and resting‐state activity. This reliable capture of obligatory sensory responses establishes a solid foundation for interpreting subsequent processing stages, as intact early sensory encoding is a prerequisite for higher‐order auditory processing.

Analyses of local effects revealed that both local stimulus types successfully elicited the MMN, validating the efficacy of the paired‐stimulus paradigm. Although classic MMN paradigms typically rely on a train of standards to establish a memory trace (Bekinschtein et al. 2009; Cowan et al. 1993; King et al. 2013; Winkler et al. 1996), our results align with the trace‐mismatch model (Naatanen 1984; Näätänen et al. 2005), where the short within‐pair ISI (150 ms) ensures that the sensory trace of the standard (S1) remains highly active for immediate comparison with the deviant (S2). This implies that the auditory system effectively detects local rule violations through this immediate predecessor relationship, without the need for long standard trains. This mechanism is supported by Yagcioglu and Ungan (2008), who applied the design of the monotonous (AB–AB–AB–AB) and the alternating (AB–BA–AB–BA) sequences to control for the neural adaptation and found significant difference waveform persisted even in paired contexts.

However, beyond this shared automatic detection mechanism, a critical dissociation emerged between simple acoustic and complex semantical deviants regarding the orienting response. Although 1500 Hz frequency‐deviants elicited MMN in 29/30 participants, they failed to trigger a robust P3a. In contrast, train‐deviants consistently evoked both MMN and P3a components with a 100% detection rate. This differential response pattern aligns with the distinction between automatic deviance detection for acoustic feature changes (Näätänen et al. 2007, 2012) and the dual‐processing model where complex semantical stimuli engage both change detection and involuntary orienting mechanisms (Escera et al. 2000). The absence of P3a for frequency‐deviants supports the stimulus complexity threshold hypothesis: Only deviants requiring higher‐order feature extraction exceed the salience threshold necessary to trigger involuntary attention shifts (Conde et al. 2016; Muller‐Gass et al. 2007). Given that cortical arousal is often diminished in DOC, basic acoustic changes may fail to breach the neural threshold required for an orienting response (Chennu and Bekinschtein 2012). In contrast, the robust P3a elicited by semantically rich information implies that such novel stimuli can effectively recruit residual attentional resources even under conditions of low arousal (Wu et al. 2020). Consequently, this neurocognitive dissociation demonstrates that train‐deviants are essential for comprehensively assessing both automatic and attention‐related processes with higher diagnostic sensitivity in clinical populations.

The global effects demonstrated clear task‐dependent modulation, providing crucial evidence for the hierarchical organization of auditory attention. In passive conditions, both global contrasts (Global_SS‐ST1_ and Global_SF‐ST2_) elicited early negativity (96–180 ms) and P3a components (196–304 ms), reflecting automatic pattern violation detection and involuntary attention orienting. However, extended positive clusters (lasting until ~400 ms) emerged only in active conditions, indicating P3b generation when voluntary attention was engaged through target counting. This selective P3b enhancement aligns with global neuronal workspace theory, suggesting that conscious access requires both bottom‐up salience and top‐down attention allocation (Dehaene et al. 2014). Notably, the similar global effect patterns between Global_SS‐ST1_ and Global_SF‐ST2_ conditions suggest that global deviance detection operates at an abstract pattern level, independent of the specific acoustic features (1000 Hz vs. 1500 Hz) that establish the global regularity.

The successful dissociation between voluntary and involuntary attention through active–passive contrasts represents a key methodological advance of our paradigm. This robust discrimination at the single‐subject level was specifically driven by the presence or absence of P3b, whereas earlier components remained remarkably stable across task conditions. The early sensory evoked potentials (P1–N1–P2 complex) showed no task‐related modulation, confirming their truly pre‐attentive nature. Specifically, the active condition exhibited an enhanced negative deflection around 200 ms and a more significant P3 compared to the passive condition. We interpret this active‐state negativity not as a “pure” MMN but likely as a composite of the automatic MMN (N2a) and the attention‐dependent anterior N2 (N2b) (Folstein and Van Petten 2008). Unlike the task‐independent MMN, the N2b component reflects voluntary processing and is elicited specifically when participants direct selective attention to deviations (Patel and Azzam 2005). Consequently, the enhanced negativity observed in our active group likely represents the superposition of this executive control component onto the automatic sensory memory trace.

Importantly, this interpretation is strongly supported by the subsequent P3a/P3b components. The robust P3b emerged selectively during active target counting, serving as confirmatory evidence for the preceding N2b. This finding aligns with previous work demonstrating that the passive condition effectively isolates involuntary processes, whereas task‐relevant processing reliably elicits voluntary attention mechanisms (Bekinschtein et al. 2009; Morlet et al. 2017, 2023). This selective modulation of the N2b/P3b provides strong evidence for the functional independence of hierarchical processing stages. Moreover, the P3b effects exhibited remarkable consistency in amplitude and morphology across the S–S and S–F global contexts. This stability indicates that under the established global rule, the brain successfully categorizes both S–S and S–F as non‐targets, thereby effectively filtering out local physical interference. Complementing this finding, the active‐passive contrast confirms that this stable P3b reflects voluntary attention rather than semantic‐driven saliency. Such precise dissociation validates our paradigm as an effective tool for probing hierarchical levels of auditory‐cognitive processing.

Our systematic evaluation of recording parameters revealed that temporal sampling outweighs spatial resolution for detecting voluntary attention. The strong positive correlation between epoch number and classification accuracy (rho = 0.97, *p* < 0.001) contrasted with the non‐significant effect of sensor density. This temporal primacy likely reflects fundamental properties of P3b generation. First, phase‐locked averaging across multiple trials progressively enhances ERP SNRs through temporal summation (Cohen 2014). Second, P3b emergence inherently requires cumulative integration across multiple cognitive cycles, as each trial contributes incremental information to reach reliable discrimination thresholds (Kok 2001; Polich 2007). Remarkably, 32‐channel configurations matched 64‐channel performance, suggesting strategic electrode placement over key generators suffices for capturing P3b dynamics. These findings indicate that clinical implementations should prioritize sufficient trial numbers over dense spatial coverage, potentially reducing setup complexity without sacrificing sensitivity.

Two specific limitations of our current design warrant consideration for future optimization. First, our between‐session comparison of active and passive conditions cannot fully exclude session‐specific effects on attention or carryover effects between conditions. A within‐session design alternating between active and passive blocks could provide more rigorous control. Second, despite successfully reducing protocol duration compared to multi‐paradigm approaches, the 40‐min session length may still challenge clinical populations with fluctuating arousal. Notably, our results suggest a potential optimization strategy: Because both Global_SS‐ST1_ and Global_SF‐ST2_ showed similar effects, the S–F pairs contributed minimally to the critical active–passive discrimination. Eliminating these pairs could reduce the paradigm to approximately 20 min without sacrificing component detection, as numerical deviants alone sufficiently elicit both local (MMN/P3a) and global (P3b) effects.

## 5 Conclusion

As demonstrated in this study, our paired‐stimulus local–global paradigm provides a comprehensive and efficient approach for assessing hierarchical auditory processing within a single experimental session. The paradigm successfully captured sequential ERP components from early sensory processing and automatic deviance detection to involuntary attention orienting and voluntary attention engagement while maintaining high individual detection sensitivity in healthy participants. Most importantly, the robust dissociation between voluntary and involuntary attention through task manipulation demonstrates that different processing levels can be selectively probed within a unified framework. Our finding that temporal sampling density outweighs spatial resolution for detecting voluntary attention offers practical guidance for optimizing recording protocols. This work establishes a methodological foundation for assessing multiple cognitive processing stages efficiently, potentially providing objective markers for evaluating residual cognitive function in behaviorally unresponsive patients.

## Author Contributions

**Chao Guo:** conceptualization, data curation, formal analysis, methodology, software, validation, visualization, writing – original draft, writing – review and editing. **Xiaoyu Wang:** conceptualization, formal analysis, investigation, methodology, supervision, writing – review and editing. **Zhaonan Ma:** conceptualization, investigation, methodology, validation. **Xiao Yang:** data curation, investigation, writing – review and editing. **Fengyu Cong:** conceptualization, funding acquisition, resources, supervision, writing – review and editing.

## Funding

This work was supported by Science and Technology Planning Project of Liaoning Province (2021JH1/10400049) and Liaoning Provincial Key Laboratory of Intelligent construction IoT Application Technology (2024KFKT‐08).

## Conflicts of Interest

The authors declare no conflicts of interest.

## Data Availability

The data supporting the findings of this research are available on request to the corresponding author, pending a formal data‐sharing agreement and approval from the local ethics committee.
